# Novel role of p73 as a regulator of developmental angiogenesis: Implication for cancer therapy

**DOI:** 10.1080/23723556.2015.1019973

**Published:** 2015-05-26

**Authors:** Maria C Marin, Margarita M Marques

**Affiliations:** 1 Instituto de Biomedicina and Departmento de Biologia Molecular; University of Leon; Leon, Spain; 2 Instituto de Desarrollo Ganadero; University of Leon; Leon, Spain

**Keywords:** angiogenesis, endothelial cell differentiation, endothelial cell migration, induced pluripotent stem cells, mouse embryonic stem cells, p73, TGF-β, vasculogenesis

## Abstract

Information regarding the role of p73 in the regulation of angiogenesis has been incomplete and quite controversial. Remarkably, several groups, including ours, have recently demonstrated that TP73 plays a fundamental role in angiogenesis regulation and that differential expression of *TP73* could have important consequences in tumor angiogenesis. Here, we discuss a possible model for p73 function in the regulation of developmental angiogenesis and tumor angiogenesis.

To address the requirement for tumor protein p73 (TP73, best known as p73) in endothelial biology we used *in vitro* physiologic models that emulate embryonic vascular development together with *in vivo* angiogenic analysis.^1-3^ Blood vessel formation is carried out by 2 tightly regulated mechanisms: vasculogenesis and angiogenesis. During development, mesodermal progenitors give rise to hemangioblasts, multipotent precursors of hematopoietic stem cells (HSCs) and angioblasts. During vasculogenesis, physiological hypoxia and proangiogenic cues induce angioblast differentiation into CD31^+^ endothelial cells (ECs) that will form a primitive vascular network.^4^ We analyzed the consequence of p73 deficiency in mouse embryonic stem cells (mESC) and induced pluripotent stem cells (iPSC) that develop into 3-dimensional cellular aggregates called embryoid bodies (EBs), in which early vasculogenesis is evident by the presence of hemangioblasts.^5^ p73 deficiency resulted in smaller EBs with a lower proportion of CD31^+^ cells, demonstrating a requirement for p73 for proper endothelial differentiation from mesodermal precursors (**Fig. 1A**). Although 2 p73 isoforms, TA and DN, were upregulated during this process, DNp73 was predominant in fully differentiated ECs, suggesting that although both isoforms are implicated in the initiation of vasculogenesis, DNp73 may have a prevailing role in differentiated ECs. In the absence of p73, primitive vascular plexus formation was severely impaired (**Fig. 1B**), with a concomitant reduction in vascular endothelial growth factor (VEGF) and transforming growth factor β (TGF-β) signaling, both of which are required for vasculogenesis.^6^ Mimicking what happens in the embryo, EBs undergo vascular remodeling by sprouting angiogenesis^5^ when a particular set of ECs loosen their cell-cell contacts, degrade the basement membrane, and become motile in response to hypoxia and proangiogenic cues (i.e., VEGF) (**Fig. 1C**). Despite addition of VEGF to the culture, p73 deficiency abrogated sprouting. Similar effects have been described in EBs upon disruption of genes such as *VEGFR2*, encoding the VEGF receptor, or *CDH5*, encoding the vascular endothelial adhesion molecule VE-cadherin. Together, these findings strongly suggest that p73 is necessary for regulation of downstream VEGF signaling, and/or EC cell-cell contact and migration.^1^

**Figure 1.**
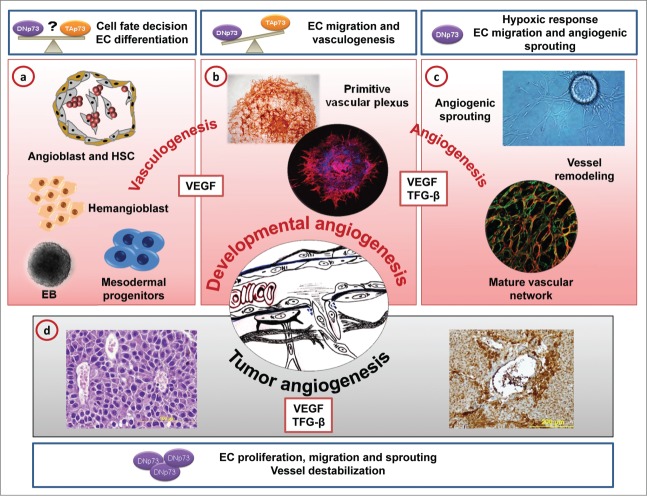
Role of p73 in developmental and tumor angiogenesis. During development, changes in the embryo microenvironment differentially regulate TAp73 and DNp73 isoforms that modulate specific differentiation programs. Thus, the cellular outcome of TA or DN p73 function will be dependent on the cellular context and will be important for cell fate determination, differentiation, vasculogenesis, and angiogenesis. (**A**) *Trp73* is required for cell fate determination and endothelial differentiation from mesodermal precursors and angioblasts. Embryonic stem cells that have differentiated into embryoid bodies (EBs) emulate vascular development. During development, mesodermal progenitors will give rise to the hemangioblast, a multipotent precursor of hematopoietic stem cells (HSC) and endothelial precursors (angioblasts). Physiological hypoxia and proangiogenic cues will induce endothelial differentiation and proliferation of angioblasts. These endothelial cells (ECs) will form a primitive vascular plexus (vasculogenesis). (**B**) In the absence of p73, formation of the plexus is impaired resulting in reduced vascular endothelial growth factor (VEGF) and transforming growth factor β (TGF-β) signaling. ECs that differentiate within the EBs form primitive vascular structures. In these cells DNp73 is the predominant isoform and regulates migration, EC-barrier establishment, and vascular plexus formation. (**C**) DNp73 is required for angiogenic sprouting, at least in part by regulating the TGF-β signaling pathway. In response to local hypoxia and proangiogenic cues (VEGF), the vascular plexus undergoes vascular remodeling by sprouting angiogenesis. Lack of p73 impairs vascular plexus maturation. (**D**) *Trp73* function is required for tumor vascularization. TAp73 deficiency might affect vessel stabilization, whereas DNp73 overexpression in tumor cells enhances their angiogenic potential.

Co-culture experiments with wild type (WT) mESCs and p73KO-iPSCs led to an intriguing observation: p73KO-iPSCs were capable of abrogating EC migration and sprout formation of WT-mESCs. These results revealed a novel non–cell-autonomous effect of p73 deficiency on angiogenic sprouting and indicated that lack of p73 resulted in the secretion of an angiogenic inhibitor. It is possible that TA and DN isoforms might be exerting an antagonistic effect over the putative inhibitor. A scenario consistent with this hypothesis has been demonstrated by Stantic and colleagues regarding p73 regulation of the expression of Bai1, a transmembrane protein that is proteolytically cleaved releasing vasculostatin, an inhibitor of EC migration.^7^

To provide genetic evidence of the angiogenic role of p73 in an *in vivo* physiological environment we used the mouse retina model to decipher the general angiogenic mechanisms that apply to developmental angiogenesis and tumor vascularization.^3^ The mouse retina starts out as an avascular tissue and ECs proliferate and migrate outwards postnatally, forming a 2-dimensional structure. This growth is directed by a network of astrocytes that, in response to the hypoxic environment, secrete VEGF. The resulting VEGF gradient polarizes ECs, which extend filopodia (tip cells) and form vascular-sprouts.^3^ p73-deficient retinas had fewer sprouts and disorganized tip cells, suggesting a defect in guidance cues. Indeed, the astrocytes had a chaotic reticulation and reduced matrix-anchored VEGF, indicating that p73 is necessary not only for EC differentiation and migration, but also to achieve the appropriate hypoxia response in non-ECs. The plexus closer to the retinal center is remodeled by pruning and secondary sprouting, generating arteries, veins, and capillary beds in which ECs establish tightly sealed lateral contacts. Lack of p73 significantly affects this process, resulting in a less branched and disorganized plexus (**Fig. 1C**). Strikingly, p73-deficient microglial cells fail to connect tip cells through their filopodia at sites of sprout anastomosis, resulting in defective network formation and supporting the role of p73 in the establishment of endothelial cell-cell contacts.

The cellular outcome of p73 activation depends on the cellular context,^8^ which determines the TA/DN p73 ratio and also the available co-factors to activate or repress specific genes. Although TAp73 and DNp73 are both upregulated during vasculogenesis, the important question that remains to be answered is how the p73 isoforms are regulated during this process. A link between the regulators of stem cell behavior and the hypoxic response during blood vessel patterning in the embryo and in the microenvironment of adult stem cell niches has been established.^9^ It is feasible to hypothesize that p73 might be differentially regulated in response to physiological oxygen changes during development and retina vascularization, especially considering the relevant role of p73 in stem cell biology. Thus, specific TA/DNp73 ratios would be necessary to bring about the full angiogenic response to hypoxia but, after the cellular response has settled down, p73 protein would be restored to basal levels. However, the nature of this regulation remains unknown. TAp73 might play a role in the endothelial differentiation process, perhaps being involved in molecular pathways that regulate expression of endothelial markers such as CD31 or VE-cadherin, or in cell fate decisions in hemangioblasts/angioblasts (**Fig. 1A**). On the other hand, our work revealed that DNp73 regulates EC migration and sprouting angiogenesis (**Fig. 1B, C**). This scenario is bolstered by the observation that DNp73 deficiency, but not TAp73 deficiency, results in an impaired endothelial morphogenesis in human umbilical vein endothelial cells (HUVECs) and affects their proangiogenic potential, demonstrating a requirement for DNp73 for EC migration and angiogenesis (**Fig. 1C**).

The proangiogenic function of DNp73 through TGFβ1/ALK1/ID1 regulation, together with its reported pro-oncogenic capacity, led us to investigate whether DNp73 overexpression augments tumor angiogenesis (**Fig. 1D**). It is noteworthy that DNp73 levels were enhanced within the tumor hypoxic microenvironment, suggesting a hypoxia-dependent regulation. As expected, DNp73 strongly boosted the angiogenic potential and proliferation of tumor cells in a syngeneic mice model, linking DNp73 overexpression to the angiogenic switch that upholds tumor progression. Moreover, 2 recent papers have thoroughly addressed the role of p73 in tumor angiogenesis.^10^ These studies link TAp73 deficiency to enhanced tumor angiogenesis and tumor progression. Stantic and colleagues showed that expression of proangiogenic factors is differentially controlled by TA and DNp73 isoforms in tumors, whereas Amelio et al. associated TAp73 overexpression with MDM2-dependent HIF1-α proteasomal degradation, reporting a concomitant downregulation of proangiogenic signaling and good patient prognosis^7,10^ and identifying TAp73 as a *bona fide* tumor suppressor gene. Thus, differential expression of p73 isoforms will be an important factor in tumor progression and might be an important target for cancer therapy.

These compounded data demonstrate for the first time the essential regulatory role of p73 in developmental angiogenesis and the important implication of deregulation of p73 isoforms in sustaining angiogenic potential and the progression of certain pathologies, such as cancer.
